# Genetic variation in the immune system and malaria susceptibility in infants: a nested case–control study in Nanoro, Burkina Faso

**DOI:** 10.1186/s12936-021-03628-y

**Published:** 2021-02-16

**Authors:** Hamatandi Magloire Natama, Eduard Rovira-Vallbona, Meryam Krit, Pieter Guetens, Hermann Sorgho, M. Athanase Somé, Maminata Traoré-Coulibaly, Innocent Valéa, Petra F. Mens, Henk D. F. H. Schallig, Dirk Berkvens, Luc Kestens, Halidou Tinto, Anna Rosanas-Urgell

**Affiliations:** 1 Department of Biomedical Sciences, Institute of Tropical Medicine, Antwerp, Belgium; 2 Unité de Recherche Clinique de Nanoro, Institut de Recherche en Sciences de La Santé, Nanoro, Burkina Faso; 3 Department of Biomedical Sciences, University of Antwerp, Antwerp, Belgium; 4 Department of Medical Microbiology-Parasitology Unit, Academic Medical Centre, Amsterdam University Medical Centres, Amsterdam, The Netherlands; 5 Centre Muraz, Bobo Dioulasso, Burkina Faso

**Keywords:** Malaria, *Plasmodium falciparum*, Immunogenetic variants, Cytokines, Innate immunity

## Abstract

**Background:**

Genetic polymorphisms in the human immune system modulate susceptibility to malaria. However, there is a paucity of data on the contribution of immunogenetic variants to malaria susceptibility in infants, who present differential biological features related to the immaturity of their adaptive immune system, the protective effect of maternal antibodies and fetal haemoglobin. This study investigated the association between genetic variation in innate immune response genes and malaria susceptibility during the first year of life in 656 infants from a birth cohort survey performed in Nanoro, Burkina Faso.

**Methods:**

Seventeen single nucleotide polymorphisms (SNPs) in 11 genes of the immune system previously associated with different malaria phenotypes were genotyped using TaqMan allelic hybridization assays in a Fluidigm platform. *Plasmodium falciparum* infection and clinical disease were documented by active and passive case detection. Case–control association analyses for both alleles and genotypes were carried out using univariate and multivariate logistic regression. For cytokines showing significant SNP associations in multivariate analyses, cord blood supernatant concentrations were measured by quantitative suspension array technology (Luminex).

**Results:**

Genetic variants in IL-1β (rs1143634) and FcγRIIA/CD32 (rs1801274)—both in allelic, dominant and co-dominant models—were significantly associated with protection from both *P. falciparum* infection and clinical malaria. Furthermore, heterozygote individuals with rs1801274 SNP in FcγRIIA/CD32 showed higher IL-1RA levels compared to wild-type homozygotes (*P* = 0.024), a cytokine whose production is promoted by the binding of IgG immune complexes to Fcγ receptors on effector immune cells.

**Conclusions:**

These findings indicate that genetic polymorphisms in genes driving innate immune responses are associated to malaria susceptibility during the first year of life, possibly by modulating production of inflammatory mediators.

## Background

Malaria is a life-threatening infectious disease caused by *Plasmodium* protozoan parasites and transmitted by *Anopheles* mosquitoes. Despite global malaria control and elimination efforts, which reduced the number of malaria-related deaths by 50% since 2000, malaria remains a major public health problem, particularly in pregnant women and children from sub-Saharan Africa [1, 2].

Individual risk for malaria infection and disease is complex and multifactorial, and is influenced/modulated by host genetic background [3, 4]. Quantitative genetics have estimated that human genetic factors could explain 25% of individual variation in susceptibility to clinical malaria in Africa [5]. An example are the numerous studies that have demonstrated a prominent role of red blood cell (RBC) polymorphisms, such as haemoglobin-inherited disorders (e.g. thalassaemia, sickle cell disease), erythrocyte membrane protein polymorphisms (e.g. ovalocytosis, spherocytosis, Duffy antigen) and erythrocyte enzymatic disorders (e.g. glucose-6-phosphate dehydrogenase) in malaria susceptibility [6, 7]. On the other hand, there is increasing evidence that identifies polymorphisms in genes related to the immune system as important determinants in susceptibility to malaria infection and disease. Immuno-genetic variants that have been associated with diverse degrees of malaria susceptibility include: (i) polymorphisms in the Human Leucocyte Antigen (HLA), which may affect recognition of parasite antigens [8–10]; (ii) polymorphisms in cytokine related genes, which may affect protein levels and down-stream functions, such as production of C-reactive protein and immunoglobulin (Ig) isotype switching [11–16]; (iii) polymorphism in toll-like receptors (TLRs), which may impair the ability of individuals to respond adequately to TLR agonists [17–21]; and (iv) polymorphisms in IgG Fcγ receptors, which may affect IgG immune complexes binding and the regulation of the IgG subclass production [22–26].

In Burkina Faso, genetic epidemiology has demonstrated that the wild-type R131 allele (rs1801274) of the FcγRIIA (CD32) and tumour necrosis factor (TNF)-238G allele (rs361525) were associated with protection from clinical malaria and high parasitaemia, respectively, in infants and children until 10 years of age [27–29]. In contrast, in a family based-study, TNF mutations rs3093664 and rs3093662 were associated with increased risk of parasitaemia and clinical malaria [27]. Overall, most of these studies have been conducted in children and adults, whereas the potential effect of immune genetic variants on infants, who are at great risk of malaria [30] and have particular immunologic characteristics (such as an immature adaptive immune system and the potential protective effect of maternal antibodies and fetal haemoglobin [31–35]), has not been investigated.

Previous studies by our group in Burkina Faso described that malaria infections and disease during the first year of life is high and has a marked age and seasonal-dependency [30], that individual heterogeneity in the risk of malaria in this age group is strongly influenced by in utero environment, with a profound effect of past placental malaria on fetal immune system [36]. The study hypothesis was that polymorphisms in genes driving Th1/Th2/innate immune response pathways may also affect the development of fetal innate immunity and thus, contribute to the heterogeneity in malaria susceptibility observed during the first year of life [30, 37, 38]. To address this question, 17 single nucleotide polymorphisms (SNPs) in 11 genes of the innate immune system previously associated with malaria-related phenotypes in African populations (including cytokines, TLRs, Fcγ receptors and nitrogen oxide synthase 2, NOS2) [4, 14, 39–42] were selected to investigated its association with malaria susceptibility in infants living in Nanoro (Burkina Faso), using a nested case–control study design.

## Methods

### Study setting

The study was conducted in the rural health district of Nanoro (Burkina Faso), located in the central-west region at 85 km from the capital Ouagadougou. Nanoro health district (NHD) has approximately 166,683 inhabitants and comprises 21 peripheral health centres. Malaria transmission is seasonal and hyperendemic with peaks between July and December and overlapping with the rainy season (June to November). Malaria burden in infants is high and strongly dependent on age and season [30]. Between 2014 and 2016, the incidence of clinical malaria was estimated at 1.03 cases per child-year during the first year of life, whereas the age-specific prevalence of asymptomatic infections ranged from 17.7% at 3 months of age to 31.3% at 12 months [30].

### Study visits and participants

A birth-cohort study (n = 734) was nested within the COSMIC clinical trial (NCT01941264) to investigate factors that modulate the risk of malaria during the first year of life [30]. COSMIC was a cluster-randomized controlled trial investigating the protective effect of adding community-scheduled screening and treatment of malaria during pregnancy (CSST) to the standard intermittent preventive treatment with sulfadoxine–pyrimethamine (CSST/IPTp-SP, intervention arm) compared to IPTp-SP alone (control arm) in Burkina Faso, Benin and The Gambia [43]. Infants from mothers participating into COSMIC trial in Burkina Faso were involved in the birth-cohort study. Infants were followed up until 12 months of age following procedures that have been described in detail elsewhere [30, 44]. In brief, malaria infections were detected actively and passively. Active case detection of asymptomatic infections consisted of 4 cross-sectional surveys conducted at 3, 6, 9, and 12 months of age. At each survey, blood films and blood spots on filter papers (Whatman 3MM, GE Healthcare Life Sciences) were collected for examination by light microscopy (LM) and quantitative real-time polymerase chain reaction (qPCR), respectively. For passive clinical case detection, mothers were encouraged to bring their offspring to the health centre if they displayed any signs of illness. Infants presenting with fever (axillary temperature ≥ 37.5 °C) on examination or history of fever in the previous 24 h were screened for malaria infection using RDT (SD-Bioline Malaria Ag P.f, Standard Diagnostic, Korea) according to manufacturer’s instructions and, if positive, treated according to national guidelines. Malaria diagnosis was subsequently confirmed by LM on Giemsa stained blood films according to standard procedures [45] and qPCR (procedure see below). For the present study, available DNA samples from 694 unrelated singletons were used for SNPs typing.

### Detection of *P. falciparum* infections by qPCR

Parasite and human genomic DNA were extracted from three punches (5 mm in diameter) of dried blood spots on filter papers with QIAamp 96 DNA blood kit (Qiagen, Germany) and eluted in 150µL of water, following the manufacturer’s recommendations. Five microliters of DNA were used as template for qPCR analysis targeting *P. falciparum var* genes acidic terminal sequence (varATS, ≈59 copies per genome) in StepOne Plus thermocycler (Applied Biosystems), as previously described [46]. The limit of detection of the varATS-qPCR was 0.1 parasite/μL as described elsewhere [47]. Samples with Ct value > 39.7 were considered negative.

### SNPs genotyping in fluidigm system

The SNPs selection for typing was performed based on existing published data. A SNP was included for analysis if: (i) had been previously associated with a malaria-related phenotype (malaria infection, clinical malaria, severe malaria, high parasites density), (ii) had an expected minor allele frequency (MAF) ≥ 5% in African populations, and (iii) had a functionally tested and/or validated TaqMan® SNPs genotyping assay. The final SNPs selected for analysis were: TLR1 (rs4833095), TLR4 (rs4986790), TLR9 (rs5743836, rs352139 and rs352140), interleukin (IL)-4 (rs2243250), IL-10 (rs1800890 and rs1800896), IL-17F (rs4715291), IL-1β (rs1143634), TNF (rs1800629 and rs3093664), interferon-γ receptor 1 (IFNR1, rs10065633 and rs10213701), nitrogen oxide synthase 2 (NOS2, rs2297518) and FcγRIIA/CD32 (rs1801274). TaqMan® SNPs genotyping assays were obtained from Applied Biosystems, and genotyping assays performed in GT192.24 dynamic arrays using the BioMark® platform (Fluidigm) at the Department of Genomics of Common Disease, Imperial College (London, UK). All samples underwent 16 cycles of specific target amplification (STA) in a total reaction volume of 10µL. Eight control samples containing known combinations of mutations at selected loci were identified from the Gambian in Western Division (GWD) population based on data from 1000 Genomes Project (http://phase3browser.1000genomes.org/), and DNA obtained from Coriell Cell Repositories. Both GWD controls and duplicate samples were included in all dynamic arrays. PCR and image processing were carried out on the BioMark® HD080 system (version 4.1.2) to determine SNP genotypes from clusters in each assay. Plots generated by the Fluidigm analysis software were subsequently visually revised to check the cluster profile for each assay and how samples with different genotypes were separated. Allelic calls for all GWD controls were consistent across all runs, and all duplicate samples within each plate had identical allele calls. The genotyping success and the overall call rate was determined per SNP.

### Haemoglobin genotyping

Haemoglobin genotypes were determined by high resolution melting (HRM) analysis, adapting previously described methods [48]. Reactions were conducted in a LightCycler® 480 System (Roche) in the presence of LightCycler® 480 HRM- Dye (HRM Master 2X, Roche Diagnostics GmbH) in a reaction of 20µL. LightCycler® 480 Gene Scanning Software (version 1.5) was used to analyse the HRM curves data and determine haemoglobin genotypes. Controls with known haemoglobin genotypes (rs 334 AA [HbAA], rs334 AT [HbAS], rs334 TT [HbSS], rs33930165 GA [HbAC]) were added to every plate.

### IL-1β and IL-1RA cytokine levels in cord blood

Levels of IL-1β and IL-1RA by cord blood cells unstimulated and stimulated with TLR7/8 agonist (a stimulant that demonstrated higher induction of cytokine production than TLR3 and TLR9 agonists in the same cohort) were determined as described previously [36]. Briefly, fresh whole cord blood samples diluted 1:1 with RPMI 1640 (1X, Gibco) were left unstimulated or stimulated with 10 µg/mL of imidazoquinoline (R848, TLR7/8 ligand; InvivoGen, San Diego, USA). Supernatant was collected following incubation for 24 h at 37 °C in 5% CO_2_, and then frozen at − 80 °C. IL-1β and IL-1RA were quantified with the fluorescent bead-based multiplex immunoassay (Human Cytokine Magnetic 30-Plex Panel kits, Novex®, Life Technologies™, USA) and analysed on a Luminex® 100/200™ instrument using Xponent 3.1 software.

### Definitions and genetic association analysis

The association between polymorphisms in immune genes and (a) malaria infection or (b) clinical malaria was investigated using case–control analysis. For malaria infection analysis, cases were infants who had at least one *P. falciparum* infection during the first year of life, regardless of symptoms; controls were infants who remained uninfected during the first 12 months of life. Uninfected infants who did not complete the 12-month follow-up were excluded from the analysis.

Clinical malaria cases were infants who experienced at least one clinical episode (presence of *P. falciparum* of any density by qPCR and an axillary temperature ≥ 37.5 °C or history of fever within the past 24 h) from birth to 12 months of age, whereas controls were who did not develop a clinical episode, irrespective of infection status.

Data was analysed using R statistical package version 3.2.3 [49]. Baseline characteristics of study participants was compared between case and control groups. Continuous data with normal distribution and categorical variables was analysed using Student *t*-test and Pearson’s chi-square tests, respectively. Hardy–Weinberg Equilibrium (HWE) was examined using Pearson’s chi-square statistical test. SNPs were excluded from the analysis if they had more or equal 10% of genotype calls missing or a significant genotypic deviation from HWE (P < 0.001). Case–control association analyses for both SNPs alleles and genotypes were assessed using logistic regression in co-dominant, dominant, over-dominant and recessive models [50]. Maternal age, gravidity, infant birth season, sex, low birth weight (LBW), prenatal malaria exposure (defined as maternal peripheral and placental infections and considered as main risk factor regardless of the preventive treatment and SP uptake received during pregnancy [36]), ethnicity and haemoglobin variants were evaluated as potential confounding factors in multivariate analyses. The crude concentrations of IL-1β and IL-1RA in un-stimulated and TLR7/8-stimulated samples were log transformed and compared using analysis of variance (ANOVA) and independent sample *t*-test. Benjamini–Hochberg method was applied to adjust *P*-values for multiple comparisons [51]. A *P*-value < 0.05 was considered statistically significant.

## Results

### Characteristics of cases and controls groups

Of the 694 infants whose DNA samples were genotyped, 656 (94.5%) completed the 12 months follow-up and were included in the analysis. Overall, 80.3% (527/656) of infants experienced at least one *P. falciparum* infection during the first year of life, out of which 78% (411/527) developed at least one clinical episode. The baseline characteristics of the study participants (cases and controls) are presented in Table 1. There were no significant differences in maternal characteristics between cases and controls for both malaria infections and clinical episodes. The proportion of infants in different categories of prenatal malaria exposure was significantly different between cases and controls for both clinical cases and malaria infection (*P* = 0.021 and *P* < 0.001, respectively). In addition, the proportion of infants born during the malaria high-transmission season was significantly higher among clinical malaria controls than cases (*P* = 0.031). In contrast, the proportion of infants born with LBW was significantly higher among clinical malaria cases than controls (*P* = 0.015). There was an ethnic homogeneity among the study participants with most infants (90%) belonging to the Mossi ethnic group. The proportion of children whose mother received 3 or more SP doses during pregnancy was significantly higher among controls than cases for both malaria infection and clinical episode (*P* = *0.006* and *P* = *0.034*, respectively). There were no significant differences in haemoglobin genotypes between cases and controls. All the participants were breast feed during the first year of life.

**Table 1:** Characteristics of study participants

Variables	Malaria infection			Clinical malaria		
	Cases (527)	Controls (129)	*P*-value	Cases (N = 411)	Controls (N = 245)	*P*-value
Maternal characteristics						
Age (years, mean ± SD)	26.5 ± 6.3	26.2 ± 6.2	0.641	26.4 ± 6.2	26.4 ± 6.3	0.988
Gravidity (no. (%))			0.176			0.250
Primigravida	88 (16.7)	25 (19.4)		69 (16.8)	44 (18.0)	
Secundigravida	92 (17.5)	14 (10.8)		74 (18.0)	32 (13.0)	
Multigravida	347 (65.8)	90 (69.8)		268 (65.2)	169 (69.0)	
MiP preventive strategy						
Standard IPTp-SP	262 (49.7)	70 (54.3)	0.355	212 (51.6)	120 (49.0)	0.520
CSST/IPTp-SP	265 (50.3)	59 (45.7)		199 (48.4)	125 (51.0)	
SP doses uptake						
< 3 doses	283 (53.7)	52 (40.3)	0.006	223 (54.3)	112 (45.7)	0.034
≥ 3 doses	244 (46.3)	77 (59.7)		188 (45.7)	133 (54.3)	
ITN usage^a^	413 (78.4)	108 (83.7)	0.152	326 (79.3)	195 (79.6)	0.934
Infant’s characteristics						
Birth season [no. in malaria high-transmission season (%)]	323 (61.3)	84 (65.1)	0.422	242 (58.9)	165 (67.4)	0.031
Gender [no. females (%)]	272 (51.6)	64 (49.6)	0.684	201 (48.9)	135 (55.1)	0.125
LBW (< 2500 g) (no. (%))	45 (8.5)	6 (4.6)	0.140	40 (9.7)	11 (4.5)	0.015
PME (no. (%))			< 0.001			0.021
Active PM	113 (23.7)	16 (13.8)		87 (23.1)	42 (19.4)	
Past PM	223 (46.8)	43 (37.0)		177 (47.1)	89 (41.0)	
MPI	53 (11.1)	19 (16.4)		47 (12.5)	25 (11.5)	
Non-exposed	88 (18.4)	38 (32.8)		65 (17.3)	61 (28.1)	
Ethnicity [no. (%)]			0.443			0.410
Mossi	471 (89.4)	122 (94.6)		366 (89.1)	227 (92.7)	
Gourounsi	52 (9.9)	6 (4.6)		42 (10.2)	16 (6.5)	
Fulani	3 (0.6)	1 (0.8)		2 (0.5)	2 (0.8)	
Samo	1 (0.2)	0 (0.0)		1 (0.2)	0 (0.0)	
Haemoglobin^b^						
AA	293 (56.8)	75 (58.6)	0.960	235 (58.3)	133 (55.2)	0.428
AC	137 (26.5)	34 (26.6)		100 (24.8)	71 (29.5)	
AS	67 (13.0)	16 (12.5)		53 (13.2)	30 (12.4)	
SC	12 (2.3)	2 (1.5)		8 (2.0)	6 (2.5)	
SS	7 (1.4)	1 (0.8)		7 (1.7)	1 (0.4)	

*PME* prenatal malaria exposure, *PM* Placental malaria, *MPI* maternal peripheral infection during pregnancy, *IPTp-SP* Intermittent preventive treatment during pregnancy with sulfadoxine–pyrimethamine, *CSST/IPTp-SP* community based-scheduled screening and treatment of malaria in addition to the standard IPTp-SP

^a^ITN usage the last night before delivery

^b^Haemoglobin genotyping was successful for 644 (98.2%) with a total of 516 infants who experienced *P. falciparum* infection while 403 developed a clinical case

### Association between immune genetic variants and malaria infection

Genotyping success per SNP was > 94% for all SNPs selected with the exception of TNF rs3093662 SNP (59.7%), which had > 10% missing data and was therefore removed from the analysis (Table 2). Call rates for all SNPs were > 96% for most assays. TLR9 rs3521140 was removed as it deviated from HWE (*P* < 0.001). Among the 15 SNPs included in the final analysis, SNPs rs4833095 (TLR1), rs3093664 (TNF-α) and rs2297518 (NOS2A) showed a minor allele frequency below 10% (Table 2).

**Table 2:** Call rate, HWE and minor allele frequencies in selected SNPs in immune genes

Gene	SNPs	Genotyping success N (%)	Overall call rate^a^	Maj/MinA	MAF	HWE (*P* value)	Malaria infection		Clinical malaria	
							MAF Cases	MAF Controls	MAF Cases	MAF Controls
TLR1	rs4833095	645 (98.3)	98.8	C/T	9.5	0.600	9.1	11.1	9.1	10.0
TLR4	rs4986790	654 (99.7)	98.4	A/G	9.0	0.999	9.2	8.1	9.1	8.8
TLR9	rs5743836	653 (99.5)	98.9	A/G	42.3	0.600	42.4	42.2	41.3	44.1
TLR9	rs352139	654 (99.7)	99.3	C/T	43.0	0.018	43.6	40.6	44.1	41.2
TLR9	rs352140	645 (98.3)	98.9	C/T	24.2	< 0.001	24.5	23.1	25.3	22.3
IL-4	rs2243250	655 (99.8)	96.4	T/C	22.1	0.600	21.8	23.3	22.3	21.6
IL-10	rs1800896	619 (94.4)	98.6	T/C	31.1	0.024	31.4	29.9	31.5	30.4
IL-10	rs1800890	650 (99.1)	97.8	A/T	20.1	0.114	20.3	19.7	21.1	18.5
IL-17F	rs4715291	655 (99.8)	99.0	C/T	15.6	0.415	16.2	13.6	16.2	14.7
IL-1β	rs1143634	654 (99.7)	99.0	G/A	11.7	0.960	10.5	16.7	10.1	14.3
TNF	rs1800629	655 (99.8)	98.8	G/A	14.5	0.961	14.1	16.0	14.4	14.7
TNF	rs3093664	656 (100)	98.9	A/G	5.6	0.600	5.5	6.2	5.2	6.3
TNF	rs3093662	392 (59.7)	97.4	A/G	5.7	–	–	–	–	–
IFNR1	rs10213701	655 (99.8)	98.6	T/A	36.4	0.863	36.5	36.0	37.3	34.8
IFNR1	rs10065633	656 (100)	99.0	T/C	50.8	0.601	51.3	48.4	51.0	50.4
NOS2A	rs2297518	656 (100)	99.4	G/A	8.2	0.180	8.5	6.6	8.4	7.8
FcγRIIA/CD32	rs1801274	648 (98.8)	98.9	G/A	32.6	0.961	30.4	41.4	29.8	37.2

*SNP* single nucleotide polymorphism, *MajA* major allele, *MinA* minor allele, *MAF* minor allele frequency, *HWE* Hardy–Weinberg equilibrium

^a^Determined by the Fluidigm SNP genotyping analysis

A first analysis investigated the association between immune genetic variants (allelic and genotypic) and *P. falciparum* infections as detected by qPCR (Table 3).

**Table 3:** SNPs included in the analysis and association with malaria infection

Gene	SNPs	Alleles or genotypes	Model	Univariate analysis		Multivariate analysis^a^	
				OR (95%CI)	*P*	AOR (95%CI)	*P*
Allele-based analysis							
TLR1	rs4833095	T vs C	–	0.80 (0.51–1.24)	0.318	0.89 (0.54–1.47)	0.660
TLR4	rs4986790	G vs A	–	1.15 (0.70–1.88)	0.581	1.44 (0.82–2.53)	0.204
TLR9	rs5743836	G vs A	–	1.01 (0.76–1.32)	0.972	0.98 (0.72–1.33)	0.760
TLR9	rs352139	T vs C	–	1.13 (0.85–1.49)	0.384	1.22 (0.89–1.66)	0.219
IL-4	rs2243250	C vs T	–	0.91 (0.66–1.27)	0.606	0.89 (0.63–1.27)	0.534
IL-10	rs1800896	C vs T	–	1.07 (0.78–1.46)	0.664	1.07 (0.76–1.53)	0.685
IL-10	rs1800890	T vs A	–	1.04 (0.73–1.46)	0.835	1.10 (0.75–1.61)	0.614
IL-17F	rs4715291	T vs C	–	1.23 (0.83–1.82)	0.305	1.11 (0.72–1.71)	0.629
* IL-1β*	*rs1143634*	*A vs G*	–	*0.58 *(*0.40*–*0.86*)	*0.006*	*0.52* (*0.34*–*0.80*)	*0.003*
TNF-α	rs1800629	A vs G	–	0.86 (0.59–1.26)	0.444	0.91 (0.60–1.38)	0.669
TNF-α	rs3093664	G vs A	–	0.88 (0.50–1.56)	0.663	0.93 (0.48–1.78)	0.826
IFNR1	rs10213701	A vs T	–	1.02 (0.77–1.35)	0.891	0.97 (0.71–1.34)	0.888
IFNR1	rs10065633	C vs T	–	1.12 (0.84–1.47)	0.407	1.20 (0.88–1.63)	0.238
NOS2A	rs2297518	A vs G	–	1.32 (0.77–2.26)	0.306	1.28 (0.71–2.33)	0.408
* FcγRIIA/CD32*	*rs1801274*	*A vs G*	–	*0.62* (*0.47*–*0.82*)	< *0.001*	*0.51* (*0.37*–*0.70*)	< *0.001*
Genotype-based analysis^b^							
TLR1	rs4833095	TT vs TC/CC	Recessive	0.60 (0.11–3.14)	0.423	0.57 (0.10–3.18)	0.521
TLR4	rs4986790	GA/GG vs AA	Dominant	1.11 (0.66–1.86)	0.828	1.43 (0.79–2.58)	0.237
TLR9	rs5743836	GG vs GA/AA	Recessive	0.87 (0.54–1.40)	0.565	0.95 (0.56–1.62)	0.861
TLR9	rs352139	TT vs TC/CC	Recessive	1.30 (0.79–2.14)	0.291	1.57 (0.89–2.77)	0.117
IL-4	rs2243250	TC vs TT/CC	Over-dominant	0.93 (0.62–1.40)	0.730	0.91 (0.58–1.43)	0.695
IL-10	rs1800896	CT vs TT/CC	Over-dominant	1.32 (0.86–2.02)	0.201	1.60 (0.99–2.61)	0.057
IL-10	rs1800890	TA vs TT/AA	Over-dominant	1.33 (0.86–2.07)	0.203	1.52 (0.93–2.48)	0.098
IL-17F	rs4715291	TT vs TC/CC	Recessive	1.15 (0.73–1.83)	0.537	1.28 (0.35–4.61)	0.710
* IL-1β*	*rs1143634*	*AG/AA vs GG*	Dominant	*0.58* (*0.37*–*0.89*)	*0.013*	*0.52* (*0.32*–*0.84*)	*0.007*
TNF-α	rs1800629	AA vs GA/GG	Recessive	1.34 (0.29–6.13)	0.704	1.41 (0.28–7.10)	0.677
TNF-α	rs3093664	GA vs GG/AA	Over-dominant	0.74 (0.41–1.35)	0.325	0.77 (0.39–1.52)	0.454
IFNR1	rs10065633	TC vs CC/TT	Over-dominant	0.89 (0.61–1.32)	0.574	0.90 (0.58–1.37)	0.619
IFNR1	rs10213701	TA vs AA/TT	Over-dominant	0.85 (0.58–1.25)	0.415	0.86 (0.56–1.32)	0.492
NOS2A	rs2297518	GA/AA vs GG	Over-dominant	1.32 (0.74–2.34)	0.342	1.30 (0.69–2.46)	0.421
*FcγRIIA/CD32*	*rs1801274*	*GA/AA vs GG*	Dominant	*0.52* (*0.34*–*0.78*)	*0.001*	*0.45* (*0.28*–*0.70*)	< *0.001*

Significant *P* values < 0.05 are indicated in italics

^a^Adjusted by mother’s age, gravidity, Birth season, baby’s sex, LBW, Prenatal malaria exposure, ethnicity, Haemoglobin variants

^b^Genotype-based analysis for dominant, over-dominant and recessive models. Models that showed lowest *P* values are shown

Both univariate and multivariate allele-based analysis showed that infants carrying the mutant allele A in IL-1β (rs1143634, adjusted odds ratio (AOR) = 0.52, 95%CI .34–0.80, *P* = 0.003) and the mutant allele A in FcγRIIA/CD32 (rs1801274, AOR = 0.51, 95%CI 0.37–0.70, *P* < 0.001) were more likely to be protected against malaria infection than infants with the wild type G allele. The genotypic analysis (univariate and multivariate) confirmed that polymorphisms in IL-1β (dominant model: AG/AA vs GG, AOR = 0.54, 95%CI 0.33–0.88, *P* = 0.014) and iFcγRIIA/CD32 (dominant model: GA/AA vs GG, AOR = 0.44, 95%CI 0.28–0.70, *P* < 0.001) were significantly associated with protection from malaria infection. Furthermore, protection was enhanced among homozygotes individuals in co-dominant models when comparing the genotypes carrying the mutant allele *versus* the wild-type genotype for both IL-1β (AA vs GG, AOR = 0.16, 95%CI 0.04–0.74, *P* = 0.019 and AG vs GG, AOR = 0.57, 95%CI 0.35–0.93, *P* = 0.026) and FcγRIIA/CD32 (AA vs GG, AOR = 0.28, 95%CI 0.14–0.55, *P* < 0.001 and GA vs GG, AOR = 0.50, 95%CI 0.31–0.81, *P* = 0.004) SNPs (Additional file 1: Table S1). On the contrary, the presence of IL-10 rs1800896 tended to increase the risk of malaria infection in over-dominant model (CT *vs* TT/CC, AOR = 1.60, 95%CI 0.99–2.61, *P* = 0.057).

### Association between immune genetic variants and clinical malaria

The allelic analysis showed that carriage of the mutant allele A in IL-1β rs1143634 SNP (AOR: 0.66, 95%CI 0.46–0.97; *P* = 0.032) and mutated allele A in FcγRIIA/CD32 rs1801274 SNP (AOR: 0.68; 95%CI 0.52–0.89; *P* = 0.005) were associated with protection from clinical malaria. The same associations were observed in the genotype-based analysis showing that individuals with AA genotype in IL-1β rs1143634 SNP (recessive model: AA vs AG/GG, AOR = 0.14, 95%CI 0.03–0.78, *P* = 0.024) and those with GA/AA genotypes in FcγRIIA/CD32 rs1801274 SNP (dominant model: GA/AA vs GG, AOR = 0.58, 95%CI 0.41–0.84, *P* = 0.003) were more likely to be protected against clinical malaria (Table 4). In the co-dominant model, the risk of clinical malaria was only reduced among homozygote AA carriers of IL-1β rs1143634 SNP (AA *vs* GG, AOR = 0.14, 95%CI 0.03–0.75, *P* = 0.022), while both AA mutant homozygote and GA heterozygote individuals for FcγRIIA/CD32 rs1801274 SNP were significantly protected (AA *vs* GG, AOR = 0.48, 95%CI 0.26–0.87, *P* = 0.017 and GA *vs* GG, AOR = 0.61, 95%CI 0.42–0.89, *P* = 0.010) (Additional file 2: Table S2). No significant association was found between the selected SNPs with increased risk of clinical malaria during the first year of life. However, a marginal increase risk was observed among infants carrying the TA genotype for rs1800890 SNP in IL-10 locus (over-dominant model: TA vs TT/AA, AOR = 1.48, 95%CI 0.99–2.19, *P* = 0.052) (Table 4).

**Table 4:** SNPs included in the analysis and association with clinical malaria

Gene	SNPs	Alleles/genotypes	Model	Univariate analysis		Multivariate analysis^a^	
				OR (95%CI)	*P*	OR (95%CI)	*P*
Allele-based analysis							
TLR1	rs4833095	T vs C	–	0.90 (0.62–1.33)	0.608	0.96 (0.63–1.46)	0.844
TLR4	rs4986790	G vs A	–	1.04 (0.70–1.54)	0.838	1.19 (0.76–1.87)	0.442
TLR9	rs5743836	G vs A	–	0.89 (0.71–1.12)	0.324	0.82 (0.64–1.06)	0.137
TLR9	rs352139	T vs C	–	1.13 (0.90–1.42)	0.296	1.09 (0.84–1.40)	0.524
IL-4	rs2243250	C vs T	–	1.04 (0.79–1.36)	0.772	0.97 (0.72–1.31)	0.857
IL-10	rs1800896	C vs T	–	1.05 (0.82–1.35)	0.676	1.00 (0.76–1.33)	0.982
IL-10	rs1800890	T vs A	–	1.17 (0.88–1.56)	0.268	1.14 (0.83–1.56)	0.423
IL-17F	rs4715291	T vs C	–	1.12 (0.82–1.53)	0.462	0.98 (0.70–9.07)	0.918
*IL-1β*	rs1143634	*A vs G*	–	*0.68* (*0.48*–*0.95*)	*0.025*	*0.66* (*0.46*–*0.97*)	*0.032*
TNF-α	rs1800629	A vs G	–	0.97 (0.70–1.33)	0.843	1.08 (0.76–1.53)	0.682
TNF-α	rs3093664	G vs A	–	0.82 (0.51–1.32)	0.406	0.73 (0.43–1.25)	0.259
IFNR1	rs10213701	A vs T	–	1.11 (0.88–1.41)	0.361	1.15 (0.89–1.50)	0.283
IFNR1	rs10065633	C vs T	–	1.02 (0.82–0.82)	0.843	1.06 (0.82–1.36)	0.644
NOS2A	rs2297518	A vs G	–	1.09 (0.72–1.65)	0.682	1.32 (0.83–2.10)	0.239
*FcγRIIA/CD32*	*rs1801274*	*A vs G*	–	*0.71* (*0.56*–*0.90*)	*0.005*	*0.68* (*0.52*–*0.89*)	*0.005*
Genotype-based analysis^b^							
TLR1	rs4833095	TT vs TC/CC	Recessive	0.44 (0.10–1.99)	0.286	0.50 (0.11–2.36)	0.388
TLR4	rs4986790	GA/GG vs AA	Dominant	1.02 (0.67–1.56)	0.910	1.28 (0.79–2.06)	0.313
TLR9	rs5743836	GG vs GA/AA	Recessive	0.81 (0.54–1.20)	0.295	0.77 (0.50–1.20)	0.250
TLR9	rs352139	TT vs TC/CC	Recessive	1.33 (0.90–1.98)	0.155	1.33 (0.86–2.06)	0.197
IL-4	rs2243250	TC vs TT/CC	Over-dominant	0.96 (0.68–1.35)	0.818	0.83 (0.57–1.20)	0.326
IL-10	rs1800896	CT vs TT/CC	Over-dominant	1.07 (0.76–1.50)	0.690	1.29 (0.88–1.88)	0.185
IL-10	rs1800890	TA vs TT/AA	Over-dominant	1.41 (0.99–2.01)	0.059	1.48 (0.99–2.19)	0.052
IL-17F	rs4715291	TT vs TC/CC	Recessive	1.29 (0.52–3.02)	0.583	0.92 (0.35–2.46)	0.882
*IL-1β*	*rs1143634*	*AA vs AG/GG*	*Recessive*	*0.29* (*0.07*–*1.18*)	*0.086*	*0.14* (*0.03*–*0.78*)	*0.024*
TNF-α	rs1800629	AA vs GA/GG	Recessive	1.34 (0.41–4.41)	0.626	2.15 (0.54–8.59)	0.279
TNF-α	rs3093664	GA vs GG/AA	Over-dominant	0.85 (0.50–1.42)	0.529	0.70 (0.40–1.25)	0.230
IFNR1	rs10065633	TC vs CC/TT	Over-dominant	1.03 (0.75–1.41)	0.869	1.14 (0.80–1.61)	0.476
IFNR1	rs10213701	TA/AA vs TT	Dominant	1.13 (0.82–1.55)	0.468	1.20 (0.84–1.70)	0.331
NOS2A	rs2297518	GA/AA vs GG	Dominant	1.11 (0.71–1.73)	0.656	1.30 (0.79–2.14)	0.307
*FcγRIIA/CD32*	*rs1801274*	*GA/AA vs GG*	*Dominant*	*0.65* (*0.47*–*0.89*)	*0.008*	*0.58* (*0.41*–*0.84*)	*0.003*

Significant *P* values < 0.05 are indicated in italics

^a^Adjusted by mother’s age, gravidity, birth season, baby’s gender, low birth weight, Prenatal malaria exposure, ethnicity, Haemoglobin variant

^b^Genotype-based analysis for dominant, over-dominant and recessive models. Models that showed lowest P values (*P)* are shown

### Association between genetic polymorphisms and cytokine levels in cord blood

**Fig. 1:**
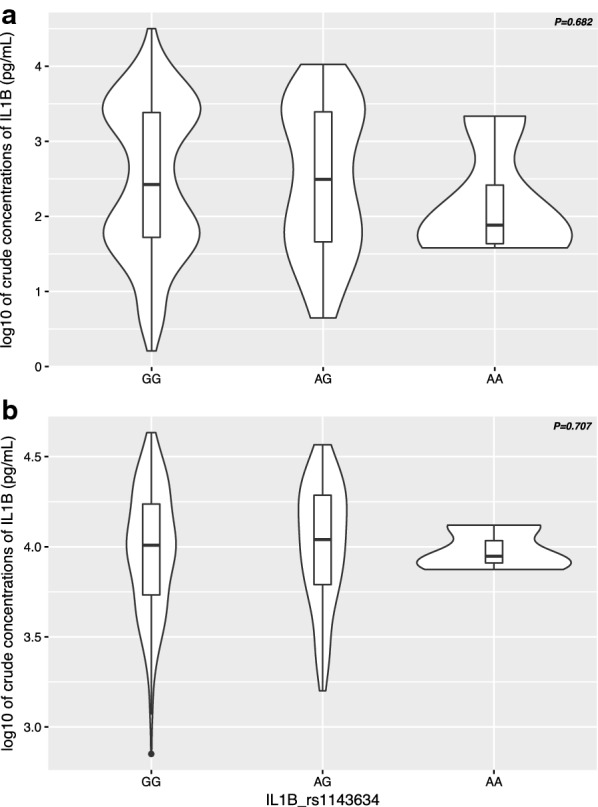
IL-1β levels in unstimulated and TLR7/8-stimulated cord blood samples across IL-1β rs1143634 SNP genotypes (GG, AG and AA). Violin plots (including median with interquartile range) showing variation of IL-1β levels across genotype groups in un-stimulated (**a**) and stimulated (**b**) samples. Numbers in each genotype group (N = 312): GG: n = 247, AG: n = 62 and AA: n = 4

Next it was assessed whether polymorphisms in IL-1β (rs1143634) were associated with immune effector phenotypes. Therefore, IL-1β levels were quantified in a sub-group of infants) for whom un-stimulated and TLR7/8-stimulated cord blood samples were available (N = 313). Figure 1 shows that IL-1β levels did not differ across rs1143634 genotypic groups for both un-stimulated and TLR-7/8-stimulated samples (*P* = 0.682 and *P* = 0.707, respectively).

**Fig. 2:**
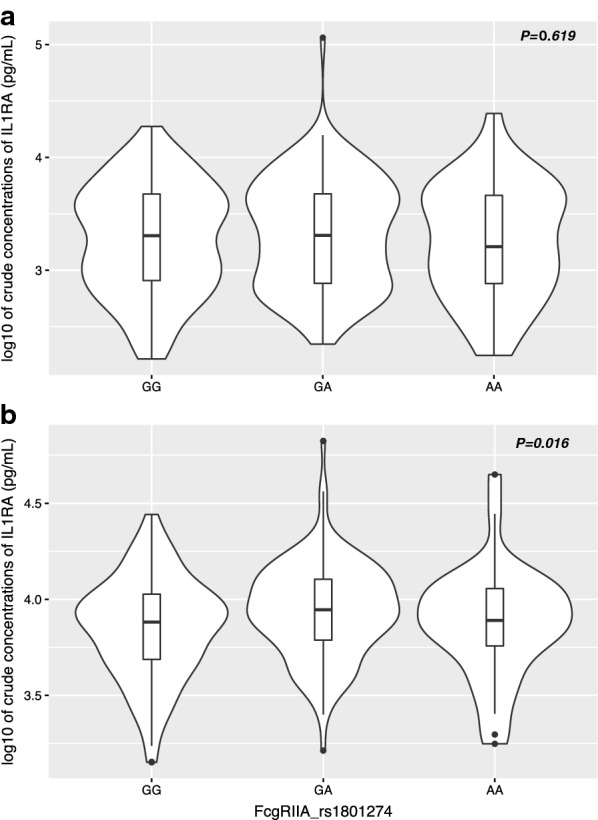
IL-1RA levels in unstimulated and TLR7/8-stimulated cord blood samples across FcγRIIA/CD32 gene (rs1801274) genotypes (GG, AG and AA). Violin plots (including median with interquartile range) showing variation of IL-1RA levels across genotype groups in un-stimulated (**a**) and stimulated (**b**) samples. Numbers in each genotype group (N = 309): GG: n = 145, GA: n = 128 and AA: n = 36

The association between FcγRIIA/CD32 rs1801274 SNP and IL-1RA levels was also investigated, since binding of FcγRIIA/CD32 to immune complexes induces IL-1RA production [52, 53]). In this case, significant differences in IL-1RA levels across FcγRIIA/CD32 rs1801274 genotypes upon TLR7/8 stimulation of cord blood samples were observed (*P* = 0.016, Fig. 2b). Individuals with GA genotype had higher levels of IL-1RA compared with that of infants carrying the wild-type GG genotype (*P* = 0.024), while no significant difference was observed for individuals carrying the AA genotype (P = 0.487). Differences of IL-1RA levels between genotypes in un-stimulated cord blood samples was not significant (*P* = 0.619, Fig. 2a).

## Discussion

This study investigated associations between genetic variants in immune genes and malaria susceptibility in infants in Burkina Faso. The study focused on cytokine, TLR, FcγRIIA/CD32 and NOS2A genes given their key role in innate immune responses against pathogens in early infancy. SNPs in FcγRIIA/CD32 (rs1801274) and in IL-1β (rs1143634) were significantly associated with protection from both malaria infection and clinical disease during the first year of life and FcγRIIA/CD32 SNP rs1801274 genotypes were associated with IL-1RA levels, a cytokine involved in inflammatory responses to malaria infection.

A number of studies have explored the association between FcγRIIA/CD32 rs1801274 SNP and malaria susceptibility resulting in contradictory findings [28, 29, 40, 54–59]. However, only a limited number included infants and the majority of those focused on severe malaria (e.g. severe malarial anaemia and high-density parasitaemia) [25, 60]. The strongest evidence of FcγRIIA/CD32 rs1801274 (A allele; variant H) protection against blood-stage malaria infections comes from a meta-analysis study on 6928 subjects from Africa and Asia [40]. Results in this study show a similar protective effect of the A allele of the FcγRIIA/CD32 rs1801274 SNP against both malaria infection and disease, with associations confirmed in both co-dominant (AA *vs* GG and GA *vs* GG) and dominant (GA/AA *vs* GG) models after adjusting by potential confounding factors.

Fcγ receptors (FcγRI, FcγRII, FcγRIII) bind to the Fc domain of IgG on immune cells and thereby mediate the initiation of a variety of immunological responses including antigen presentation, phagocytosis, cytotoxicity, release of inflammatory mediators and the modulation of immune responses [61, 62]. Therefore, FcγRs are an important link between cellular and humoral immunity in host defence against malaria infection [62]. FcγRIIA, which is expressed on the surface of all types of cells of the immune system, is a low affinity receptor for monomeric IgG, but binds IgG immune complexes efficiently [63]. FcγRIIA/CD32 rs1801274 has been found to alter the gene function in vitro, by extending the preferential affinity of the wild-type G allele for IgG1 and IgG3 towards affinity for IgG2 [57, 64]. Therefore, it’s reasonable to believe that the efficient binding to IgG2 while retaining its affinity to IgG1 and IgG3 increase the antibody mediated protective effect against malaria. It has been also suggested that differential binding between immune complexes and Fcγ receptors due to polymorphisms in FcγRIIA induce changes in the ability of immune cells to respond to *P. falciparum* infection through production of inflammatory mediators [58, 62, 64]. Here, the effect of rs1801274 SNP in FcγRIIA/CD32 gene on unstimulated and TLR-stimulated IL-1RA production in cord blood samples was tested, as it has been previously demonstrated that FcγRIIA/CD32 plays a key role in IL-1RA production through binding of immune complexes to monocyte, macrophage and neutrophil lineage cells [52, 53]. Several studies have demonstrated the clinical relevance of IL-1RA in immune responses to malaria [65–68], which acts modulating disease severity by competing with the pro-inflammatory cytokines IL-1α and IL-1β for binding sites on the IL-1 type I receptor and inhibits IL-1 signalling [69]. While there were no significant differences in the spontaneous production of IL-1RA by cord blood cells across FcγRIIA/CD32 rs1801274 genotypes, upon TLR7/8 stimulation, individuals with GA genotype had significantly higher levels of IL-1RA compared with those with the wild type GG genotype. Remarkably, in infants from this cohort TLR7/8-induced IL-1RA production in cord blood was an independent predictor of malaria protection during the first year of life [36]. Therefore, study findings support the hypothesis that variability in susceptibility to malaria across FcγRIIA/CD32 rs1801274 genotypes is mediated, at least partially, by changes in immune complex-mediated cytokine production. In addition to the protective effect of IgG1 and IgG3, FcgR2A mutant carriers are likely to be protected due to the high affinity of the A allele product to IgG2 possibly by improving the antibody mediated protective effect. Given that this protective effect could be effective through the mediation of a variety of immunological responses including inflammatory mediators, level of IL1-RA could represent one of the changes in innate immune response to malaria infection between the mutant A and the wild-type G allele carriers. The fact that a significant difference was not observed for homozygous allele could be due to limited number of individuals carrying the AA genotype in the subset of data analysed.

The second genetic variant in immune genes associated with malaria protection in this study was the IL-1β rs1143634 SNP. The carriage of IL-1β rs1143634 mutated allele A (both in homozygosity and heterozygosity) was associated with a decreased risk of malaria infection (84% and 43% reduction, respectively). In addition, AA homozygote carriers (but not AG) had a significantly reduced risk of clinical malaria, suggesting an enhanced protective effect of AA homozygosity. This is the first study to report a protective effect of IL-1β rs1143634 SNP against malaria infection and uncomplicated disease in infants. Previous studies assessing associations of IL-1β rs1143634 and malaria susceptibility reported heterogeneous findings in children including an association with higher peripheral parasitaemia in Ghanaian children (n = 461; aged 1–12 years) [70], an association with severe malaria in Gambian children (n = 1420; aged < 5 years) [71], or no significant association with malaria susceptibility in Cameroonian children (n = 1862; aged 1–14 years) [72]. These contrasting results may be attributed to differences in age groups, ethnicity and/or malaria phenotype evaluated.

IL-1β is a pro-inflammatory cytokine that is implicated in the first line of defence against pathogens, including the hepatic and erythrocytic stages of malaria parasites [73–75]. However, high levels of sustained IL-1β production may induce pathogenic effects that promote disease manifestation and severity [76–78]. Previous studies have already demonstrated a correlation between IL-1β rs1143634 SNP and increased in vitro production of IL-1β [79, 80]. To investigate whether the observed protective effect of the rs1143634 SNP in this study was related to functional changes in IL-1β production, IL-1β levels in cord blood were compared across genotypic groups but no significant differences were observed neither in un-stimulated or TLR7/8-stimulated samples. Genetic variations occurring in genes encoding inflammatory cytokines can have a direct effect on the innate immune responses to malaria infection and disease manifestation. In this regard, the protective effect of the IL-1β rs1143634 could be due to the modulation of IL-1β level towards a protective effect instead to levels that could be harmful.

However, these findings should be interpreted with caution due to very low number of individuals carrying the AA genotype among the subset of infants that could be analysed (n = 4) and, therefore, potential functional changes in IL-1β production by IL-1β rs1143634 SNP cannot be ruled out. Likewise, it is worth noting that IL-1β production is likely to be influenced by other SNPs in IL-1β gene, as well as polymorphisms in inflammatory mediators such as TNF [81–84] that were not assessed in this study. Correlation between SNP of interest and cytokine levels in different genotypes should be further explored in different malaria phenotypes (severe, uncomplicated, infection) in the field to elucidate these contrasting results.

Other genes coding for major TLRs (i.e. TLR1, 4 and 9) and cytokines (i.e. IL-4, IL-10, IL-17F and TNF) did not show significant association with malaria infection and clinical episodes, although rs1800890 SNPs in IL-10 gene showed a trend towards increased susceptibility to malaria infection or clinical episode in multivariate analysis (*P* = 0.057 and *P* = 0.052, respectively). IL-10 is a key immuno-regulator of immunity to infections [85] and has been associated with various malaria-related phenotypes, such as uncomplicated malaria, severe malaria, severe malaria anaemia and high parasite density [86–91]. In addition, a number of studies have reported associations between IL-10 SNPs and susceptibility to malaria with either protective effects in some SNPs and increased susceptibility in others [72, 92–95]. Therefore, this tendency of an increased risk of malaria among carriers of rs1800890 SNP in IL-10 gene, could results from changes that do not allow adequate modulation of inflammatory responses since IL-10 is an anti-inflammatory cytokine.

## Conclusion

This study showed that the mutated A allele of IL-1β rs1143634 and mutated A allele of FcγRIIA/CD32 rs1801274 are associated with protection against both malaria infections and uncomplicated disease during the first year of life. These findings suggest that genetic polymorphisms in genes driving innate immune responses could condition malaria susceptibility during the first year of life, possibly by modulating production of inflammatory mediators. Future investigations on the epistatic effects of immune genes polymorphisms described here and functional studies may contribute to further understand how host genetic variation influences malaria susceptibility in infancy.

## Supplementary Information

**Additional file 1: Table S1.** Genotypic-based association analysis for malaria infection using co-dominant models.

**Additional file 2: Table S2.** Genotypic-based association analysis for clinical malaria using co-dominant models.

## Data Availability

The datasets during and/or analysed during the current study available from the corresponding author on reasonable request.
