# Supplementary material for: Genetic variation in the immune system and malaria susceptibility in infants: a nested case–control study in Nanoro, Burkina Faso
Source: Malar J. 2021 Feb 16;20:94. doi: 10.1186/s12936-021-03628-y (PMC7885350; doi:10.1186/s12936-021-03628-y)
Supplement: Supplementary file 1 — Additional file 1: Table S1. Genotypic-based association analysis for malaria infection using co-dominant models. [file 12936_2021_3628_MOESM1_ESM.docx]

**Table S1. Genotypic-based association analysis for malaria infection using the co-dominant model**

| **Gene** | **SNPs** | **Genotypes** | **Univariate analysis** | | **Multivariate analysis*** | |
| --- | --- | --- | --- | --- | --- | --- |
|  |  |  | **OR (95%CI)** | ***P*** | **OR (95%CI)** | ***P*** |
| TLR1 | rs4833095 | TC vs CC | 0.81 (0.49-1.35) | 0.423 | 0.94 (0.54-1.66) | 0.845 |
| TLR1 | rs4833095 | TT vs CC | 0.58 (0.11-3.04) | 0.521 | 0.56 (0.10-3.16) | 0.515 |
| TLR4 | rs4986790 | GA vs AA | 1.06 (0.63-1.78) | 0.828 | 1.37 (0.76-2.48) | 0.298 |
| TLR4 | rs4986790 | GG vs AA | - | - | - | - |
| TLR9 | rs5743836 | GA vs AA | 1.19 (0.77-1.84) | 0.431 | 0.98 (0.61-1.57) | 0.935 |
| TLR9 | rs5743836 | GG vs AA | 0.96 (0.56-1.63) | 0.876 | 0.94 (0.52-1.71) | 0.847 |
| TLR9 | rs352139 | TC vs CC | 0.99 (0.64-1.52) | 0.967 | 0.94 (0.59-2.85) | 0.814 |
| TLR9 | rs352139 | TT vs CC | 1.30 (0.75-2.25) | 0.351 | 1.53 (0.82-2.85) | 0.183 |
| IL-4 | rs2243250 | TC vs TT | 0.92 (0.60-1.39) | 0.687 | 0.90 (0.57-1.42) | 0.653 |
| IL-4 | rs2243250 | CC vs TT | 0.87 (0.40-1.88) | 0.718 | 0.85 (0.36-2.00) | 0.714 |
| IL-10 | rs1800896 | CT vs TT | 1.31 (0.84-2.04) | 0.235 | 1.55 (0.93-2.57) | 0.089 |
| IL-10 | rs1800896 | CC vs TT | 0.96 (0.52-1.77) | 0.888 | 0.83 (0.42-1.63) | 0.591 |
| IL-10 | rs1800890 | TA vs AA | 1.29 (0.83-2.03) | 0.256 | 1.47 (0.89-2.42) | 0.132 |
| IL-10 | rs1800890 | TT vs AA | 0.71 (0.32-1.58) | 0.402 | 0.70 (0.30-1.62) | 0.408 |
| IL-17F | rs4715291 | TC vs CC | 1.18 (0.74-1.86) | 0.486 | 1.06 (0.64-1.75) | 0.819 |
| IL-17F | rs4715291 | TT vs CC | 1.64 (0.47-5.65) | 0.435 | 1.29 (0.36-4.71) | 0.695 |
| **IL-1β** | **rs1143634** | **AG vs GG** | **0.62 (0.39-0.96)** | **0.032** | **0.57 (0.35-0.93)** | **0.026** |
| **IL-1β** | **rs1143634** | **AA vs GG** | **0.27 (0.70-1.02)** | **0.053** | **0.16 (0.04-0.74)** | **0.019** |
| TNF-α | rs1800629 | GA vs GG | 0.78 (0.51-1.21) | 0.273 | 0.85 (0.53-1.36) | 0.501 |
| TNF-α | rs1800629 | AA vs GG | 1.25 (0.27-5.78) | 0.768 | 1.34 (0.26-6.81) | 0.720 |
| TNF-α | rs3093664 | GA vs AA | 0.75 (0.41-1.36) | 0.339 | 0.78 (0.39-1.53) | 0.467 |
| TNF-α | rs3093664 | GG vs AA | - | - |  |  |
| IFNR1 | rs10065633 | TC vs TT | 1.00 (0.63-1.60) | 0.993 | 1.07 (0.64-1.80) | 0.782 |
| IFNR1 | rs10065633 | CC vs TT | 1.25 (0.73-2.16) | 0.415 | 1.42 (0.79-2.58) | 0.239 |
| IFNR1 | rs10213701 | TA vs TT | 0.88 (0.59-1.34) | 0.562 | 0.88 (0.56-1.38) | 0.577 |
| IFNR1 | rs10213701 | AA vs TT | 1.18 (0.61-2.26) | 0.620 | 1.08 (0.54-2.14) | 0.831 |
| NOS2A | rs2297518 | GA vs GG | 1.29 (0.71-2.33) | 0.399 | 1.28 (0.66-2.47) | 0.464 |
| NOS2A | rs2297518 | AA vs GG | 1.78 (0.22-14.63) | 0.591 | 1.56 (0.18-13.43) | 0.683 |
| **FcγRIIA/CD32** | **rs1801274** | **GA vs GG** | **0.56 (0.36-0.85)** | **0.007** | **0.50 (0.31-0.81)** | **0.004** |
| **FcγRIIA/CD32** | **rs1801274** | **AA vs GG** | **0.40 (0.22-0.74)** | **0.004** | **0.28 (0.14-0.55)** | **<0.001** |

*Adjusted by mother’s age, gravidity, Birth season, baby’s sex, LBW, Prenatal malaria exposure, ethnicity, Haemoglobin variants
