# Supplementary material for: Genetic variation in the immune system and malaria susceptibility in infants: a nested case–control study in Nanoro, Burkina Faso
Source: Malar J. 2021 Feb 16;20:94. doi: 10.1186/s12936-021-03628-y (PMC7885350; doi:10.1186/s12936-021-03628-y)
Supplement: Supplementary file 2 — Additional file 2: Table S2. Genotypic-based association analysis for clinical malaria using co-dominant models. [file 12936_2021_3628_MOESM2_ESM.docx]

**Table S2. Genotypic-based association analysis for clinical malaria using co-dominant models**

| **Gene** | **SNPs** | **Genotypes** | **Univariate analysis** | | **Multivariate analysis*** | |
| --- | --- | --- | --- | --- | --- | --- |
|  |  |  | **OR (95%CI)** | ***P*** | **OR (95%CI)** | ***P*** |
| TLR1 | rs4833095 | TC vs CC | 0.99 (0.65-1.53) | 0.991 | 1.11 (0.69-1.77) | 0.670 |
| TLR1 | rs4833095 | TT vs CC | 0.44 (0.10-1.99) | 0.286 | 0.51 (0.11-2.40) | 0.398 |
| TLR4 | rs4986790 | GA vs AA | 1.01 (0.66-1.54) | 0.979 | 1.28 (0.79-2.09) | 0.308 |
| TLR4 | rs4986790 | GG vs AA | 1.79 (0.18-17.25) | 0.614 | 1.08 (0.10-11.11) | 0.948 |
| TLR9 | rs5743836 | GA vs AA | 0.96 (0.67-1.37) | 0.820 | 0.91 (0.62-1.35) | 0.656 |
| TLR9 | rs5743836 | GG vs AA | 0.79 (0.50-1.23) | 0.301 | 0.73 (0.45-1.20) | 0.220 |
| TLR9 | rs352139 | TC vs CC | 0.95 (0.67-1.36) | 0.794 | 0.94 (0.63-1.39) | 0.758 |
| TLR9 | rs352139 | TT vs CC | 1.30 (0.83-2.02) | 0.247 | 1.29 (0.79-2.10) | 0.308 |
| IL-4 | rs2243250 | TC vs TT | 0.98 (0.69-1.38) | 0.895 | 0.84 (0.57-1.22) | 0.356 |
| IL-4 | rs2243250 | CC vs TT | 1.20 (0.61-2.35) | 0.595 | 1.09 (0.53-2.24) | 0.808 |
| IL-10 | rs1800896 | CT vs TT | 1.08 (0.76-1.54) | 0.650 | 1.26 (0.85-1.87) | 0.240 |
| IL-10 | rs1800896 | CC vs TT | 1.07 (0.63-1.79) | 0.804 | 0.90 (0.51-1.59) | 0.717 |
| IL-10 | rs1800890 | TA vs AA | 1.40 (0.97-2.01) | 0.067 | 1.46 (0.98-2.18) | 0.064 |
| IL-10 | rs1800890 | TT vs AA | 0.92 (0.45-1.87) | 0.811 | 0.86 (0.41-1.81) | 0.696 |
| IL-17F | rs4715291 | TC vs CC | 1.09 (0.75-1.59) | 0.633 | 1.04 (0.69-1.58) | 0.848 |
| IL-17F | rs4715291 | TT vs CC | 1.32 (0.53-3.30) | 0.552 | 0.94 (0.35-2.49) | 0.899 |
| IL-1β | rs1143634 | AG vs GG | 0.73 (0.50-1.07) | 0.111 | 0.82 (0.54-1.25) | 0.357 |
| **IL-1β** | **rs1143634** | **AA vs GG** | **0.27 (0.68-1.11)** | **0.070** | **0.14 (0.03-0.75)** | **0.022** |
| TNF-α | rs1800629 | GA vs GG | 0.91 (0.63-1.31) | 0.615 | 0.93 (0.63-1.38) | 0.722 |
| TNF-α | rs1800629 | AA vs GG | 1.31 (0.40-1.31) | 0.656 | 2.11 (0.52-8.45) | 0.293 |
| TNF-α | rs3093664 | GA vs AA | 0.84 (0.50-1.42) | 0.519 | 0.70 (0.39-1.25) | 0.232 |
| TNF-α | rs3093664 | GG vs AA | 0.58 (0.08-4.17) | 0.592 | 1.29 (0.11-15.30) | 0.838 |
| IFNR1 | rs10065633 | TC vs TT | 1.05 (0.71-1.55) | 0.804 | 1.17 (0.76-1.80) | 0.479 |
| IFNR1 | rs10065633 | CC vs TT | 1.04 (0.67-1.62) | 0.843 | 1.06 (0.65-1.70) | 0.826 |
| IFNR1 | rs10213701 | TA vs TT | 1.09 (0.78-1.53) | 0.617 | 1.20 (0.83-1.75) | 0.336 |
| IFNR1 | rs10213701 | AA vs TT | 1.27 (0.76-2.14) | 0.357 | 1.16 (0.66-2.02) | 0.603 |
| NOS2A | rs2297518 | GA vs GG | 1.11 (0.70-1.77) | 0.643 | 1.31 (0.78-2.20) | 0.313 |
| NOS2A | rs2297518 | AA vs GG | 1.01 (0.24-4.26) | 0.991 | 1.20 (0.27-5.27) | 0.806 |
| **FcγRIIA/CD32** | **rs1801274** | **GA vs GG** | **0.68 (0.48-0.95)** | **0.026** | **0.61 (0.42-0.89)** | **0.010** |
| **FcγRIIA/CD32** | **rs1801274** | **AA vs GG** | **0.53 (0.31-0.90)** | **0.020** | **0.48 (0.26-0.87)** | **0.017** |

*Adjusted by mother’s age, gravidity, Birth season, baby’s sex, LBW, Prenatal malaria exposure, ethnicity, Haemoglobin variants
